# Bipolar Disorder: Clinical Perspectives and Implications with Cognitive Dysfunction and Dementia

**DOI:** 10.1155/2012/275957

**Published:** 2012-05-28

**Authors:** R. Lopes, L. Fernandes

**Affiliations:** ^1^Psychiatry Service, Hospital de São João (HSJ), EPE, 4200-319 Porto, Portugal; ^2^Faculty of Medicine, University of Porto (FMUP), 4200-319 Porto, Portugal

## Abstract

*Introduction*. Cognitive dysfunction as a core feature in the course of bipolar affective disorder (BPD) is a current subject of debate and represents an important source of psychosocial and functional burden. *Objectives*. To stand out the connection and clinical implications between cognitive dysfunction, dementia, and BPD. *Methods*. A nonsystematic review of all English language PubMed articles published between 1995 and 2011 using the terms “bipolar disorder,” “cognitive dysfunction,” and “dementia”. *Discussion*. As a manifestation of an affective trait or stage, both in the acute phases and in remission, the domains affected include attention, executive function, and verbal memory. The likely evolution or overlap with the behavioural symptoms of an organic dementia allows it to be considered as a dementia specific to BPD. This is named by some authors, as BPD type VI, but others consider it a form of frontotemporal dementia. It is still not known if this process is neurodevelopmental or neurodegenerative in nature, or both simultaneously. The assessment should consider the iatrogenic effects of medication, the affective symptoms, and a neurocognitive evaluation. *Conclusion*. More specific neuropsychological tests and functional imaging studies are needed and will assume an important role in the near future for diagnosis and treatment.

## 1. Introduction

For a long time mild and severe cognitive deficits have been recognized as part of mental disorders. In affective disorders, although initially considered as secondary features related to social or psychological factors, they are now recognized as playing an integral part of the clinical expression [1] and a connection with dementia has also been suggested [2]. More than half of patients with prolonged BPD over 60 years have cognitive deficits [3] and about two-thirds have subjective amnesic complaints [1].

Yet, the recognition of the impact of these deficits on psychosocial and occupational functioning [4–6] and therapeutic compliance (patients often attribute these deficits to medication side effects) is an important feature of BPD [7]. Also, it certainly contributes to an impairment of functional recovery, which even in euthymia is achieved slowly, despite modern symptomatic treatments [7]. In fact, recent reports have documented that some cognitive deficits (processing speed and verbal learning/memory) are independent predictors of functional recovery [6, 8] and also that improvements in neurocognitive status could predict changes in functional outcome [9].

The consideration of cognitive impairment as a neurodevelopmental or neurodegenerative/progressive process is a controversial issue that remains under discussion today and the most accepted view is that it is probably a combination of both etiopathogenic mechanisms. In cases of dementia, further doubts remain if neurocognitive impairment could be a marker of progressive decline to a specific dementia of BPD, but the coexpression with other types of organic dementia could also be a possibility. Therefore, it is not definitely known whether this is a chance association or if there is an etiopathogenic link between BPD and dementia [10].

## 2. Clinical Features

### 2.1. Cognitive Dysfunction

In BPD, the cognitive dysfunction probably has a multifactorial aetiology involving a gene-environment interaction. There is remarkable interindividual variability in its clinical functioning [8]. In general, there are changes in the areas of verbal memory, attention, and to some extent in executive function and visual memory [11]. Although remission occurs in euthymia in the visual memory and working memory domains, the persistence of the others may be a marker for progression to a neurodegenerative process [7, 12]. This is somewhat contrary to the assumption of Emil Kraepelin that full recovery existed between episodes in BPD.

In fact, results of recent meta-analytic studies have documented the existence of cognitive deficits in all phases of BPD, which are apparently independent of the affective state [11, 13]. These involve selective attention/processing speed, concentration, immediate episodic memory, attentional deviance, strategic thinking [7, 12], abstraction, verbal learning/immediate memory/planning, and perseveration [1, 11, 13–19]. Also, the deficits in executive function (initiation of response and inhibitory control) are probably related to dysfunction of the prefrontal cortex and may be responsible for reducing the patient's coping abilities, making individuals more vulnerable to recurrence of symptoms [20]. Notwithstanding, there is relative preservation in the visuospatial memory, verbal fluency, and vocabulary [21–23].

In studies of emotional processing, both the perception of identity and the recognition of facial affection were found to be preserved in the acute phases or in remission [20]. With regard to social cognition, the studies addressing the Theory of Mind (ToM, e.g., the ability to represent one's own and others' mental states) have showed that a probable interaction with other cognitive domains such as attention or poor executive functioning may be involved [20]. A recent report, however, concluded that impairment on ToM is partially independent of dysfunction in other cognitive domains and the mood state, and even may represent a trait marker of BPD [24]. Parts of the widespread neural network involving cortical midline structures in ToM are in accordance with the neuroanatomical and neuroimaging findings, namely, insula, temporoparietal junction, anterior cingulate cortex, and precuneus [25]. However, the existing data is still scarce.

### 2.2. Cognitive Dysfunction in BPD Type I, BPD Type II, and Unipolar Depression

Most studies refer to BPD type I, which is more associated with verbal memory, visual memory, and semantic fluency deficits; BPD type II patients, on the other hand, probably have less widespread and severe cognitive dysfunction than BPD type I [11]. This is somewhat in accordance with the association of psychosis or mania events and more severe impairment in the memory domain, which is more common in BPD I. In fact, BPD patients with a past history of psychosis showed more severe deficits in verbal memory [18, 26, 27] and working memory/executive function [28] than those without a history. There is an association between BPD type I patients with a positive family history of psychotic illness and worse performance in selective attention and visual-motor processing [29]. A common pathophysiology in the medial temporal regions to verbal memory and semantic fluency deficits for a potentially specific endophenotype of BPD type I was proposed [30], in accordance with neuroimaging findings [31].

Also, there seem to be differences in the cognitive deficits domains related to type of affective state. Mania is associated with more pronounced deficits in verbal memory and executive function, while depression is associated (albeit with less statistical power) with deficits in executive function, verbal learning, and visual and spatial memory. Interestingly, an initial episode of mania in early ages (rather than a depressive episode) was associated with increased risk of late cognitive dysfunction [4]. Also, in depressive and untreated episodes (compared with those treated), there are pronounced deficits in visual recognition of facial expressions and attention [18, 26, 27] and also poorer verbal fluency [32]. It is not known to date if a more severe profile in BPD type I patients is due to the neurotoxic effects of manic episodes or due to neurobiological differences from the onset of illness.

The cognitive deficits between bipolar and unipolar depression appear similar, but more severe in the first [33]. A recent study, although with a short sample, also concluded for similar psychosocial and neurocognitive functioning among major depressive disorder (MDD) and BPD patients during a depressive episode, in the case of severe and complex mood disorders [34].

Apparently, there is a positive association between psychotic symptoms, age of onset (the number of affective episodes associated with decreased motor speed and executive function) [35], and duration of the disease (associated with decreased verbal memory) [4, 18] with severity of cognitive deficits [19] and also with the risk of a diagnosis of dementia [22]. An association to environmental factors, namely, obstetric complications, infection with herpes simplex virus type 1 (HSV-1), and early traumatic adversity was also documented [36].

It is generally agreed that BPD is pleomorphic, with individual heterogeneous presentations and different outcomes. Some patients experience a worse clinical course from the beginning, while others have good interepisodic functional recoveries despite multiple recurrences. Notwithstanding, there are still doubts whether a poorer course is the consequence of cognitive deficits, which is more unfavourable than previously thought and whether this is stable and developmental or neurodegenerative/progressive in nature [37].

Progressive and stage-related neuroanatomical changes and cognitive decline are in concordance with progressive biochemical changes [38]. These occur not only in the well-documented monoamine and second messenger abnormalities but also in inflammatory cytokines, corticosteroids, neurotrophins, mitochondrial energy generation, oxidative stress, and neurogenesis [39]. Taking into account this progressive nature of BPD, known as neuroprogression, a staging model for BPD was proposed by Berck and collaborators [40, 41]. In this model, the clinical stage is based on the assumption that in earlier stages of the disease, as opposed to chronic ones, there are better prognosis and better response to treatment [40]. This can also be viewed as a course specifier, in which an early diagnosis and intervention seems tangential with the parallel notion of neuroprotection [13, 38]. The progressive nature of evolution of BPD could therefore be reversible with the appropriate algorithm treatment comprising this neuroprotective measure and/or novel agents [38, 40]. Furthermore, progressive shortening of the inter-episodic interval, cognitive impairment and higher rates of physical comorbidity and mortality during the course of BPD are in accordance with reduced probability of treatment response [38]. This highlights the importance of effective long-term prophylaxis, because it seems that resilience decreases with the cumulative effects of chronic stress and intermittent episodes. This effect was proposed as the allostatic load in BPD, in which recurrent stress induces abnormalities in the brain that lead to changes in processing information [42]. Consequently, a greater number of recurrent episodes and stressors make patients more vulnerable or less resilient to subsequent episodes or stressors [43].

Although it seems that cognitive functioning deteriorates over time with disease progression and worsens with repeated acute episodes, there are few longitudinal studies addressing the neurocognitive function and outcome. The findings of a prospective longitudinal study over 3 years to investigate the stability and specificity of cognitive impairment in BPD type I, after controlling for age and length of illness, suggest this is mainly stable over time [44]. But, in a follow-up study over 15 years, cognitive dysfunction, namely in processing speed and verbal learning domains, was independently associated with social and global functioning outcome in BPD [6]. The different nature of both processes could rather explain the differences between BPD type I and type II neurocognitive profile [30]. Although it seems that cognitive deficits are present in the first episode [45], there is a lack of studies of premorbid functioning evaluating possible alterations that occurred before the establishment of the disorder.

### 2.3. A Specific Dementia of BPD?

This topic is currently under discussion and some authors have suggested different views. Akiskal and colleagues suggest a particular form of late-onset of BPD in the elderly, accompanied by cognitive dysfunction, named by the authors as BPD type VI, in previously healthy individuals. According to these authors there is a concomitant clinical interface of mood instability, irritability and aggression with disturbance of memory and other cognitive deficits in an early onset of dementia [46]. Thus, nonspecific behavioural symptoms of dementia can be an expression with an affective episode of a co-morbid undiagnosed BPD, or otherwise to promote the expression of latent bipolarity [46, 47]. For another perspective, a pre-existing BPD undiagnosed can also be postulated as contributing to the symptoms of affective dysregulation of dementia [48]. This is an important perspective to consider as BPD represents approximately 20% of mood disorders in the elderly [49] and 8% of new cases of BPD occur in geriatric patients [50]. Also, the spectrum of BPDs in the general population is 5.4 to 8.3% [51], so a high number of patients will remain without a diagnosis.

So, the hypothesis of the coexistence of an organic dementia and a bipolar spectrum disorder represents an alternative to the neurological clinical view that agitation, impulsivity and mood instability of dementia are an expression of frontal lobe dysfunction [52]. In fact, the psychopathological similarities between the behavioural manifestations of dementia and affective episodes of BPD have become a real clinical challenge for their diagnosis and proper treatment. Low social engagement, fatigue and lack of initiative can be present in a depressive state and can be common between the two entities, a situation that can lead to diagnostic confusion. Disorders of the sleep-wake cycle and delusional activity of similar types are common in both entities. Thus, an inappropriate motor behaviour in dementia can be a form of disorganized hyperactivity of BPD. Denial and anosognosia of dementia can be understood as the omnipotence of mania, and expensive spending of mania can be interpreted as an altered behaviour of dementia. Familiarity, joy and loss of social conformity can be a component of frontal dysfunction and also be secondary to the elation of humour. In fact, the evidence of relative similarity with frontal lobe dysfunction, documented in changes of personality, apathy and social disability in addition to the type of cognitive domains involved and neuroimaging findings (see next section) has led some authors to propose the dementia of BPD as a form of clinical presentation of a frontal-subcortical dementia (frontotemporal dementia-FTD) [53]. Besides that, there seem to be differences in the frontal signs, observed by less self-neglect and less emotional indifference and also differences related to outcome and prognosis [53]. A possible pathological connection process between schizophrenia and BPD reported in a series of cases of FTD supports the hypothesis of these two pathologies being linked in the same cerebral region. Additionally, it suggests a subgroup within the frontotemporal lobar degeneration connected to the outcome of dementia in some bipolar patients [54]. Interestingly, psychiatric symptoms can be a prodrome or an independent risk factor in patients who go on to develop neurodegenerative disease (ND). A recent study involving 252 patients in a speciality clinic, 28,2% with a diagnostic ND, received a prior psychiatric diagnosis. The patients with the behaviour variant frontotemporal dementia were at the highest risk to misdiagnosis (52.2%) and were more likely to receive diagnosis of BPD or schizophrenia, than the other dementia groups. This was positively associated with a younger age, higher education, and a family history of psychiatric illness [55]. The significant symptom overlap between dementia and primary psychiatric disorders is a particular diagnostic challenge. It remains, however, difficult to establish the true nature of these dementia disorders despite data emerging from neuropsychological and neuroimaging studies.

### 2.4. Genetic Studies

 Mutations in genes related to migration and neurodevelopment were identified in a subgroup of bipolar and schizophrenic patients, which predicted the seriousness of prefrontal cognitive deficits in both disorders. Also, a developmental delay in verbal memory and executive domains was demonstrated in paediatric BPD [56]. However, most appear to fit into a neurodegenerative model, which further contributes to medical comorbidities, impaired psychosocial functioning, the number of episodes, and biological changes [42]. Also, polymorphisms in brain-derived neurotrophic factor and cathecol O-methyltransferase (COMT) were associated with abnormal cognitive function [57, 58]. An additive gene-environment effect has been documented with polymorphisms of COMT and seropositivity to HSV-1 [59].

In family studies, deficits in executive function and verbal memory [22] and also cognitive flexibility and attentional shift [21] were demonstrated in healthy siblings. The fact that they are very similar to those described in the euthymic phase suggests that they may be genetic vulnerability markers or traits, again supporting the neurodevelopmental hypothesis. Also, some cognitive deficits appear to be influenced by the same genes that predispose to illness [60]. But that fact is not entirely clear, since there are no conclusive prospective studies to assert which and in what gravity (or risk-enhancing factors) can be predictors to development of different types of affective disorders [61] and their impact on functional status.

### 2.5. Neurochemical, Neurophysiological, Neuroanatomical, and Neuroimaging Studies

High concentrations of regional brain monoamine substances [62], abnormally increased glucocorticoid receptor function [63], and high concentrations of homocysteine [64] were found in neurochemical studies. In euthymic patients an increase in amplitude of waves was found on electroencephalogram, especially in brain areas associated with visuospatial processing deficit [65].

As occurred in unipolar depression, cognitive deficits may be related to structural brain abnormalities, and several studies have found that the latter can probably be predictors of their development and/or even of dementia [1]. It was reported that patients with BPD could have brain tissue loss, although the speed of grey matter loss was slow and well correlated with the deterioration of cognitive function and mood episodes [66].

The changes of parenchyma seem not to be pervasive but region specific. In fact, the most significant ones refer to increase in the hippocampus and in amygdala volume, mild ventricular enlargement (predominant in the right lateral ventricle), and changes in association cortex (prefrontal cortex, anterior cingulate and dorsolateral prefrontal nucleus) [67]. Importantly, the changes at the level of the cingulate cortex (atrophy of frontal and temporal lobes) seem to predispose to frontal and temporal circuit dysfunction. This is in accordance with predominant hypoperfusion at frontotemporal regions observed in studies of functional neuroimaging [67]. In a recent quantitative meta-analysis of functional magnetic ressonance imaging (fMRI) studies in BPD, there was abnormal frontal-limbic activation [68]. This was manifested by underactivation of the inferior frontal cortex or ventrolateral prefrontal cortex (consistent across emotional and cognitive tasks and particularly related to the state of mania) and overactivation of limbic areas (including medial temporal structures: parahippocampal gyrus, hippocampus, and amygdala) and basal ganglia [68]. These last were elicited by emotional but not cognitive tasks and also not clearly related to mood states [68]. This is somewhat in corroboration with the findings of a recent investigation using diffusion tensor tractography in BPD patients which found specific white matter fiber bundle abnormalities and disrupted integrity connecting structures of the anterior limbic network [69].

Also, studies with brain MRI revealed the existence of white matter hyperdensity of periventricular and deep subcortical location associated with cognitive deficits in prolonged illness and also with a poor prognosis [70]. However, white matter lesions in euthymic patients with cognitive impairment did not differ from asymptomatic controls [71]. The lack of alterations in cerebral morphology in elderly euthymic BPD patients could suggest either a progressive neurotoxic process [72]. Again, because abnormalities in volume of the amygdala have been found similar in both children and adults, and also in patients with first or multiple episodes, functional neuroanatomical deficits do not necessarily reflect a neurodegenerative process [42].

## 3. Evaluation

### 3.1. General Aspects

The rigorous evaluation of cognitive deficits apparent on clinical examination in BPD requires a systematic approach to psychiatric and medical comorbidities, iatrogenic effects, and a neuropsychological evaluation. Firstly it is necessary to consider the patient's age (independent risk factor) as well as the intelligence quotient and premorbid level of education. Compared with BPD adult patients, geriatric BPD patients have a similar pattern of cognitive dysfunction, but children with BPD [73] show the greatest effects in verbal memory and the smallest differences in motor speed and intelligence [74].

In cases of dementia where there is a suspicion of an unrecognized bipolar spectrum disorder, the difficulty in the evaluation makes help from family a valuable way of getting information. Prior psychiatric history (episodes of mania and/or depression, suicide attempts, anxiety disorders, alcoholism, and substance abuse), previous personality (cyclothymic, or irritable, or hyperthymic temperament) [46, 47], and life-events (disturbances in interpersonal relationships, unemployment, etc.) are important elements that should be addressed in the history assessment. Premorbid personality described as energetic and prone to hyperactivity [46] and a positive family history of BPD are important associations in patients with comorbid dementia and late-onset BPD [47, 48].

### 3.2. Psychiatric and Medical Comorbidities

Anxiety disorder, obsessive compulsive disorder, dissociative disorder, substance abuse disorder, and other aspects of psychopathology are often comorbid with BPD [75]. Its evaluation becomes important because changes in these psychopathological states can alter cognitive functions such attention and processing speed [11, 76]. Also, a positive association of severe cognitive dysfunction with diabetes [4] and other high prevalent medical comorbidities such as cerebrovascular disease in this population has been documented (24% in long-term illness have two or more risk factors) [77].

### 3.3. Iatrogenic Effects

Cognition can be modified by iatrogenic or drug interactions often inherent in polypharmacy [36, 78]. As a potential aggravating and confounding factor, this is important to take into account in view of avoiding untimely antidementia prescription, instead of reduction, replacement, or elimination of drugs that may be the cause of such symptoms.

The use of mood stabilizers requires an analysis of risk/benefit. Changes in cognitive function secondary to the use of lithium are well documented in healthy subjects and euthymic patients and are independent of the disease. Reduction of short-term memory, motor function, verbal associative fluency, and attention [11] is documented and patients typically report feeling of dullness and decreased cognitive creativity. Hypothyroidism resulting from chronic use of this drug may also be associated with apathy and cognitive deficits.

The range of cognitive deficits expected with the use of other mood stabilizers is diverse being virtually absent with lamotrigine and gabapentin [79–81]. These are relatively modest and transient (in learning and memory domains) with sodium valproate and carbamazepine. A diffuse effect particularly in attention, verbal memory, psychomotor retardation, and difficulties in the recall of words is documented with topiramate [82, 83].

Most studies addressing cognitive function with antipsychotics refer to schizophrenia and, in BPD, apart of being scarce, were carried out in a small number of patients. However, there is documented adverse effects at the level of planning tasks and processing speed [84], and also on executive function [85]. Despite inconsistencies, the use of benzodiazepines is associated with problems in working memory and processing speed, and its prolonged use could confound neuropsychological assessment [86]. Antidepressants, with the exception for anticholinergic effects of tricyclics [87] do not have important documented adverse cognitive effects, and, on the contrary, recent evidence shows that serotonin may have a positive effect on working memory [88].

### 3.4. Neuropsychological Evaluation

Given the heterogeneity of clinical expression of cognitive dysfunction in BPD, it is necessary to carry out a thorough neuropsychological evaluation. Along with the Minimental State of Examination, it must include tests that analyse the domains of attention, memory, and executive function, among others [11]. There is not at present a battery of neuropsychological tests adapted and validated for BPD. This will be an important support to the clinical decision on treatment, rehabilitation, and restoration of functional capacity adapted to the real world in the future [89]. Moreover, it is an important vehicle for investigation of neuroanatomical and functional changes, genetic phenotypes, and expression of symptoms and clinical benefits or disadvantages of treatment.

The promising results of a battery of cognitive tests for clinical research in the area of cognitive dysfunction in schizophrenia (measurement and treatment research to improve cognition in schizophrenia: MATRICS Consensus Cognitive Battery, MCCB) [90–92] recently served as a model for research in cognitive assessment in BPD [20]. This initiative promoted by the International Society for Bipolar Disorders intended to identify in the literature the cognitive tests included in the MCCB best adapted to BPD, taking into account the fact that cognitive deficits are similar in pattern but less severe than in schizophrenia [11] and also the overlapping aspects of neurobiology, genetics, risk factors, and neuropsychological functioning. This investigation concluded that the tests included in the MCCB (concerning processing speed, attention/vigilance, working memory, verbal learning, visual learning, executive function, and social cognition) should be complemented by more complex tests at the level of verbal learning (California Verbal Learning Test) and executive function (Stroop Test, Trail Making Test—part B, Wisconsin Card Sorting Test) [20]. Also, it will include specific tests for emotional processing and social cognition of BPD [20]. This is in accordance with the results of a recent study using the MCCB in BPD type I patients, where a statistically significant impairment was verified in five of seven MCCB domains, but in less impairment on the reasoning and problem-solving and social cognition domains [93]. Nevertheless, and although more studies are needed to test for repeatability, this may be a feasible instrument in cognitive trails for BPD [93].

## 4. Treatment

Treatment of cognitive dysfunction in BPD requires the determination of specific cognitive deficits, aiming its etiological origin [11] and the thymic stabilization of the disorder [36]. The lack of specific pharmacological therapy that can substantially improve the cognitive symptoms remains currently under investigation, but drugs with a favourable or neutral cognitive profile are advisable.

The treatment of behavioural and psychological symptoms of dementia and a possible concomitant bipolar spectrum disorder should be more focused on mood stabilizers than antidepressants. This is because of antidepressants' iatrogenic effects inducing conversion to excitability, hypomania, or mania. In the context of dementia this elation of mood can be expressed by a worsening of behavioural and psychological symptoms. Apparently, antidepressants may be refractory and even worsen behavioural symptoms [47]. Thus, the onset of agitation in dementia, after use of antidepressants, should assume the investigation of a bipolar spectrum disorder.

Evidence of use of mood stabilizers on BPD in the elderly is scarce [94], and in general, the maximum doses are lower and require a slower titration. Lithium is rarely prescribed for the first time after the age of 70 because of its narrow therapeutic index and greater risk of neurotoxicity and other side effects with age [48, 95]. However, neuroprotective effects of mood stabilizers, as opposed to chronic stress in neurons, are also described [96]. As they can also have a role in reducing the number of recurrences, these are indirect ways of preventing dementia [49].

The effectiveness of sodium valproate, in addition to being better tolerated than carbamazepine, is well documented in the treatment of behavioural and psychological symptoms of dementia and also in behavioural and sleep disturbances in patients with mania [97, 98]. Dorey and collaborators have proposed the valproate as the mood stabilizer of choice [48] with a gradual dose titration of about 125–250 mg/day to a maximum dose of 500–1000 mg/day [94]. Antipsychotics may be considered only in situations of agitation associated with another mood stabilizer [99], and although there is evidence of benefit [47], its use requires caution and is even contraindicated in most cases of dementia.

The utility of procholinergic drugs (donepezil, galantamine, rivastigmine) in behavioural symptoms of dementia is well documented. Their probable usefulness in cognitive dysfunction is related to their action on rostral and basal cholinergic forebrain pathways and in the frontal-striatal dopaminergic tone. Therefore they may have a role in attention, working memory [100], and information processing [101]. Only modest improvement of memory was verified in AD, and in schizophrenia, it did not have demonstrable benefits [102, 103]. In BPD, in particular, despite the limited and reduced number of studies available, improvement was verified with donepezil [104], and possible cognitive benefits with galantamine [105, 106]. Notwithstanding, as cases of mania in patients with dementia and comorbid BPD have been reported in literature with their utilization, caution with its use should be observed [107].

Relative to antiglutamate agents, data available refers particularly to schizophrenia, and although the majority (memantine, d-cycloserine, and glycine) had no benefit over placebo [108, 109], there is evidence of improvement in executive function and in global cognitive functioning with d-serine [110]. However, one study showed potential efficacy of memantine in the improvement of global cognitive function, although with a reduced sample [111].

Another area of research is focused in understanding the aetiology of hypercortisolism associated with mood disturbances, which justifies the interest in the glucocorticoid receptor antagonists. In fact, the use of mifepristone (RU-486), 600 mg/day, in patients with BPD was associated with improvement in spatial working memory, verbal fluency, spatial recognition memory, and depressive symptoms (although in a small number of participants) [112].

Psychostimulants (amphetamine, methylphenidate) seem to be potentially attractive in the syndrome of attention deficit and hyperactivity, apathy of MDD, Parkinson's disease, craneoencefalic trauma, and apathy of AD [113]. However, the available data is scarce, and it is necessary to meet its side effects [114].

Modafinil may be also a wake-promoting agent to be taken into account in the future since its use was associated with improvement in attention, memory, executive function, visual memory, and recent verbal immediate recall in chronic schizophrenia [11, 115].

The potential use of antiparkinsonian drugs such as pramipexole, pergolide, ropinirole, bromocriptine, and amantadine results of the D2/D3 agonist effects at the frontal lobe and consequently on the level of executive processing. In fact, there was an improvement of cognitive function in a study using amantadine and pergolide in healthy subjects, and in another study using pramipexole added to mood stabilizing agents in the context of treatment of the depressive phase of BPD patients [116].

The usefulness of antioxidants such as L-Carnosine has been advocated in the prevention of formation of free radicals in lipid peroxidation of the neuronal membrane and excitoxicity of glutamate. These adverse effects on BPD originate a reduced neuronal viability (documented by a reduction in more than 40% of the enzyme glutamate decarboxylase and the enzyme superoxide dismutase within the hippocampus, and increased toxic activity of glutamate in the synaptic clefts in mania episodes) [117]. The identification of progressive oxidative, inflammatory, and neurotrophic abnormalities associated in parallel with stage-related structural and neurocognitive alterations advocates the development of novel treatment classes with a neuroprotective effect [39].

The nonpharmacological strategies should be defined by a regular follow-up and monitoring of affective and cognitive symptomatology and the therapeutic instituted, in a collaborative effort of the various specialties involved (general medicine, neurology, and psychiatry). Prevention and treatment of cerebrovascular risk factors, particularly with changing lifestyles such as exercise and healthy diet including vitamins and mineral supplements, become equally essential in the treatment process.

It is becoming essential to carry out systematic studies of strategies of psychotherapy addressing cognitive dysfunction on BPD [95], as well as cognitive training and rehabilitative strategies in cognitive domains most predictive of long-term functional outcome. There is in the last field a nonrandomized pilot study utilizing cognitive remediation showing some improvements in executive functioning [118].

## 5. Conclusions

Cognitive dysfunction and dementia associated with BPD is still a subject little studied. It is related to psychosocial and functional impairment and also to lower treatment compliance, representing an important therapeutic target in the future.

The identification of specific cognitive deficits in the domains of attention, verbal memory, learning, and executive functions both during acute stages and in clinical remission has become important in the clinical approach, but there is still a lack of a relationship between cognitive function and psychosocial outcome across illness phases. A neurodegenerative and/or neurodevelopmental process involving genetic and environmental risk factors probably combines to play a role and further studies are needed to elucidate this process. The determination of a specific dementia in BPD, with a neuropsychological and imaging profile similar to, but with better outcomes than an FTD dementia type, needs to be clarified. In this context, a detailed clinical history of bipolar traits (premorbid personality, psychiatric and family history) is useful to their differential diagnosis, but even a chance association between the two entities cannot be excluded.

In this sense, it is imperative to promote further research in order to obtain instruments for neuropsychological evaluation specifically targeted for the diagnosis, to the best understanding of the nature and severity of cognitive deficits. A subsequent differentiation of bipolar subgroups, especially concerning psychotic symptoms, along with their neuropsychological profile could be important in the establishment of the outcome, which is still to be determined as stable or progressive. The importance of development of preventive strategies and proper treatment could be used in modifying the prognosis of the disease and in the improvement of functional recovery rates.

On the other hand, further studies are needed to clarify the extent of negative side effects of psychotropic drugs on cognition and assessment of its risk/benefit ratio and also its relationship with psychosocial functioning. Notwithstanding, the clinician should be aware of the drugs with better cognitive profile as well as the effective treatments for comorbid conditions. Although there is currently no specific pharmacotherapy target to cognitive dysfunction in patients with BPD, new agents such as modafinil or mifepristone constitute potential options for the future. The worsening of behavioural symptoms with the use of antidepressants in situations of coexistence of BPD and dementia reorients treatment strategies particularly for the use of mood stabilizers.

The authors expect with this work to have made a contribution in understanding the connection between dementia and BPD, which, in the light of present knowledge, needs to be further investigated with prospective studies and functional imaging.
